# Randomized comparison of low dose cytarabine with or without glasdegib in patients with newly diagnosed acute myeloid leukemia or high-risk myelodysplastic syndrome

**DOI:** 10.1038/s41375-018-0312-9

**Published:** 2018-12-16

**Authors:** Jorge E. Cortes, Florian H. Heidel, Andrzej Hellmann, Walter Fiedler, B. Douglas Smith, Tadeusz Robak, Pau Montesinos, Daniel A. Pollyea, Pierre DesJardins, Oliver Ottmann, Weidong Wendy Ma, M. Naveed Shaik, A. Douglas Laird, Mirjana Zeremski, Ashleigh O’Connell, Geoffrey Chan, Michael Heuser

**Affiliations:** 1 Department of Leukemia, University of Texas, MD Anderson Cancer Center, Houston, TX USA; 2 Otto-von-Guericke University Medical Center, Magdeburg, Germany; 3 Department of Haematology and Transplantology, Medical University of Gdańsk, Gdańsk, Poland; 4 Department of Hematology and Oncology, University Hospital Hamburg-Eppendorf, Hamburg, Germany; 5 Johns Hopkins Sidney Kimmel Comprehensive Cancer Center, Baltimore, MD USA; 6 Department of Hematology, Medical University of Lodz, Lodz, Poland; 7 Hospital Universitari i Politècnic La Fe, Valencia, Spain; 8 CIBERONC, Instituto Carlos III, Madrid, Spain; 9 Division of Hematology, University of Colorado School of Medicine, Aurora, CO USA; 10 Hôpital Charles LeMoyne, Greenfield Park, QC Canada; 11 Division of Cancer and Genetics, School of Medicine, Cardiff University, Cardiff, UK; 12 Pfizer Oncology, New York, NY USA; 13 Department of Hematology, Hemostasis, Oncology and Stem Cell Transplantation, Hannover Medical School, Hannover, Germany; 14 Present Address: Internal Medicine II, University Hospital Jena, Jena, Germany

**Keywords:** Acute myeloid leukaemia, Molecularly targeted therapy

## Abstract

Glasdegib is a Hedgehog pathway inhibitor. This phase II, randomized, open-label, multicenter study (ClinicalTrials.gov, NCT01546038) evaluated the efficacy of glasdegib plus low-dose cytarabine (LDAC) in patients with acute myeloid leukemia (AML) or high-risk myelodysplastic syndrome unsuitable for intensive chemotherapy. Glasdegib 100 mg (oral, QD) was administered continuously in 28-day cycles; LDAC 20 mg (subcutaneous, BID) was administered for 10 per 28 days. Patients (stratified by cytogenetic risk) were randomized (2:1) to receive glasdegib/LDAC or LDAC. The primary endpoint was overall survival. Eighty-eight and 44 patients were randomized to glasdegib/LDAC and LDAC, respectively. Median (80% confidence interval [CI]) overall survival was 8.8 (6.9–9.9) months with glasdegib/LDAC and 4.9 (3.5–6.0) months with LDAC (hazard ratio, 0.51; 80% CI, 0.39–0.67, *P* = 0.0004). Fifteen (17.0%) and 1 (2.3%) patients in the glasdegib/LDAC and LDAC arms, respectively, achieved complete remission (*P* < 0.05). Nonhematologic grade 3/4 all-causality adverse events included pneumonia (16.7%) and fatigue (14.3%) with glasdegib/LDAC and pneumonia (14.6%) with LDAC. Clinical efficacy was evident across patients with diverse mutational profiles. Glasdegib plus LDAC has a favorable benefit–risk profile and may be a promising option for AML patients unsuitable for intensive chemotherapy.

## Introduction

Myelodysplastic syndrome (MDS) and acute myeloid leukemia (AML) are clinically and genetically heterogeneous myeloid stem cell disorders with a median age at onset of about 67 years [1]. Older patients with AML or high-risk MDS have few treatment options and are often not eligible for intensive chemotherapy due to comorbidities and a higher incidence of high-risk biological features, which often lead to chemotherapy resistance.

This population is thus treated with less-aggressive therapies, including low-dose cytarabine (LDAC) and hypomethylating agents. However, studies with LDAC have demonstrated low response rates (7–18%), with median overall survival (OS) of 5 months in older patients [2–7]. With the hypomethylating agent decitabine, the response rate (18%) and median OS (7.7 months) were only slightly improved [5]. Therefore, novel therapeutic strategies are needed to achieve higher response rates, more durable responses, and improved survival in this hard-to-treat population.

The Hedgehog signaling pathway plays a key role in embryonic development and is typically silenced in adults [8]. Aberrant Hedgehog signaling has been implicated in hematologic malignancies and is critical for leukemia stem-cell survival and expansion [9–11]. Overexpression of Hedgehog pathway components was observed in chemotherapy-resistant myeloid leukemia cells, and pharmacologic inhibition of the Hedgehog pathway substantially enhanced the sensitivity to chemotherapy [12]. These findings provide the rationale for combining an inhibitor of Hedgehog pathway with chemotherapy.

Glasdegib is a potent and selective oral inhibitor of Hedgehog signaling through binding to Smoothened. In preclinical studies, glasdegib produced rapid and complete tumor regression as a single agent or in combination with chemotherapy, reduced expression of key leukemia stem-cell regulators, and decreased leukemia stem-cell populations in patient-derived AML cells [13, 14]. Glasdegib monotherapy demonstrated preliminary clinical activity in phase I trials in patients with hematologic malignancies [15, 16]. Therefore, glasdegib plus chemotherapy represents a mechanistically attractive treatment approach for patients with AML or MDS.

A phase Ib/II, open-label, international, multicenter study evaluated safety and efficacy of glasdegib plus intensive chemotherapy (cytarabine and daunorubicin), LDAC, or decitabine in previously untreated patients with AML or high-risk MDS [17, 18]. Here we describe results from the ongoing phase II, randomized, open-label portion of the study that assessed the efficacy and safety of glasdegib plus LDAC (glasdegib/LDAC) versus LDAC in patients with AML or high-risk MDS who were not eligible for intensive chemotherapy.

## Methods

### Patients

Eligible patients were aged ≥55 years with newly diagnosed, previously untreated AML or high-risk MDS according to the World Health Organization (WHO) 2008 Classification [19]. For a diagnosis of high-risk MDS RAEB-2 (refractory anemia with excess blasts 2), the patient must have 10–19% bone marrow blasts. Patients had to have a known cytogenetic profile at study entry and considered not suitable for intensive chemotherapy, defined by ≥1 of the following criteria [20]: age ≥75 years; serum creatinine >1.3 mg/dL, severe cardiac disease (e.g., left ventricular ejection fraction <45% by multi-gated acquisition or echocardiography at screening), or Eastern Cooperative Oncology Group Performance Status (ECOG PS) = 2. Patients with ECOG PS = 0 or 1 who met ≥1 other inclusion criteria listed above were also eligible (for full inclusion criteria, see Supplementary materials). Patients were excluded if they had acute promyelocytic leukemia, t(9;22) cytogenetic translocation, active other malignancy, known active uncontrolled leukemia of the central nervous system, or prior treatment with Hedgehog inhibitor or other investigational agent for the treatment of an antecedent hematologic disease (for more details, see Supplementary materials).

### Study design and treatment

In this open-label, multicenter phase II study (ClinicalTrials.gov, NCT01546038) carried out in Europe and North America, patients were stratified by cytogenetic risk factor (good/intermediate or poor) and randomized (2:1) to receive glasdegib/LDAC or LDAC. The primary objective was OS. Secondary objectives included clinical efficacy endpoints, safety and tolerability, pharmacokinetics (PK), pharmacodynamics, and effect on corrected QT (QTc) interval.

Patients were classified as having poor-risk disease if they had one of the following cytogenetic features: inv(3), t(6;9), 11q23, –5, –5q, –7, abnormal (17p), or complex karyotype (≥3 clonal abnormalities). Patients with none of these features were classified as having good/intermediate-risk disease [21, 22]. Glasdegib 100 mg once daily was administered orally in 28-day cycles on a continuous basis and LDAC 20 mg was administered subcutaneously twice daily for 10 days every 28 days. Patients remained on treatment until disease progression, unacceptable toxicity, or patient refusal. All patients were followed up for post-treatment survival status for 4 years from randomization.

Patient randomization was obtained by the investigator or the designee from the interactive voice response system. Masking was not applicable for this open-label study.

This study was conducted in compliance with the Declaration of Helsinki, the International Council for Harmonisation Good Clinical Practice Guideline, and local regulatory requirements. The final protocol, amendments, and informed consent documents were approved by institutional review board or independent ethics committee at each investigational center. All patients provided informed consent.

### Assessments

#### Efficacy

Response to treatment was assessed based on the International Working Group response criteria and WHO Guidelines for MDS and AML [23, 24]. Immunophenotyping and cytogenetics were performed for all bone marrow samples (Supplementary materials).

#### Pharmacokinetics

Blood samples for PK analysis of glasdegib were analyzed for concentrations of glasdegib at Covance Bioanalytical Services, LLC (Indianapolis, IN, USA) using a validated, sensitive, and specific high-performance liquid chromatography–tandem mass spectrometric approach (Supplementary materials).

#### Safety

Safety assessments included adverse events (AEs), classified and graded based on the National Cancer Institute Common Terminology Criteria for Adverse Events v4.0, laboratory evaluations, vital signs, physical examinations, and 12-lead electrocardiograms. Treatment duration and time of treatment exposure of glasdegib were also calculated (Supplementary materials).

#### Biomarker analyses

Biomarker assessments included mutational status of the following genes: *CEBPA*, *DNMT3A*, *FLT3*, *IDH1*, *IDH2*, *KIT*, *KRAS*, *NPM1*, *NRAS*, *RUNX1*, *TET2*, and *WT1*. Whole blood samples from serial blood draws were analyzed for gene expression using TaqMan Low-Density Microarrays, including 21 target genes implicated in Hedgehog pathway signaling and/or AML pathobiology (Supplementary materials).

#### Statistical analyses

OS was defined as time from the date of randomization to death from any cause. Patients not known to have died at the last follow-up were censored on the date they were last known to be alive. The reported median OS for LDAC was approximately 5 months [2–4] and the expected median OS for glasdegib/LDAC was 8 months, resulting in an expected hazard ratio (HR) = 0.625 (i.e., 60% improvement in OS). A total of 132 patients would be randomized at 2:1 ratio (i.e., 88 in the glasdegib/LDAC arm and 44 in the LDAC alone arm), of which 92 OS events observed would provide 80% power to detect the 60% improvement in OS at one-sided significance level of 0.10 with an interim analysis (IA) for futility. The IA would occur when 46 OS events were observed (i.e., 50% information). Since the IA was for futility only, no alpha would be spent at the IA. The rho(1) spending function was used as the beta-spending function for futility at the IA. If exactly 46 OS events were observed at the IA, the futility boundary would be crossed if the observed HR > 0.92. The futility boundary would be calculated accordingly using the chosen spending function and number of OS events actually observed at the IA.

Median OS and 80% confidence interval (CI) were analyzed using the Kaplan–Meier method. A stratified log-rank test (one-sided *α* = 10%) was used to compare OS between the treatment arms. A Cox proportional hazard regression stratified by prognosis (good/intermediate versus poor) was used to estimate the HR and 80% CI of OS. Other efficacy endpoints were summarized descriptively and included complete remission (CR) and CR with incomplete blood count recovery (CRi). An additional efficacy endpoint for AML included morphologic leukemia-free state (MLFS). Additional efficacy endpoints for MDS included marrow CR (mCR) and partial remission. Safety data were summarized descriptively and included all randomized patients who received at least one dose of any of the study medications.

## Results

### Patients

**Fig. 1:**
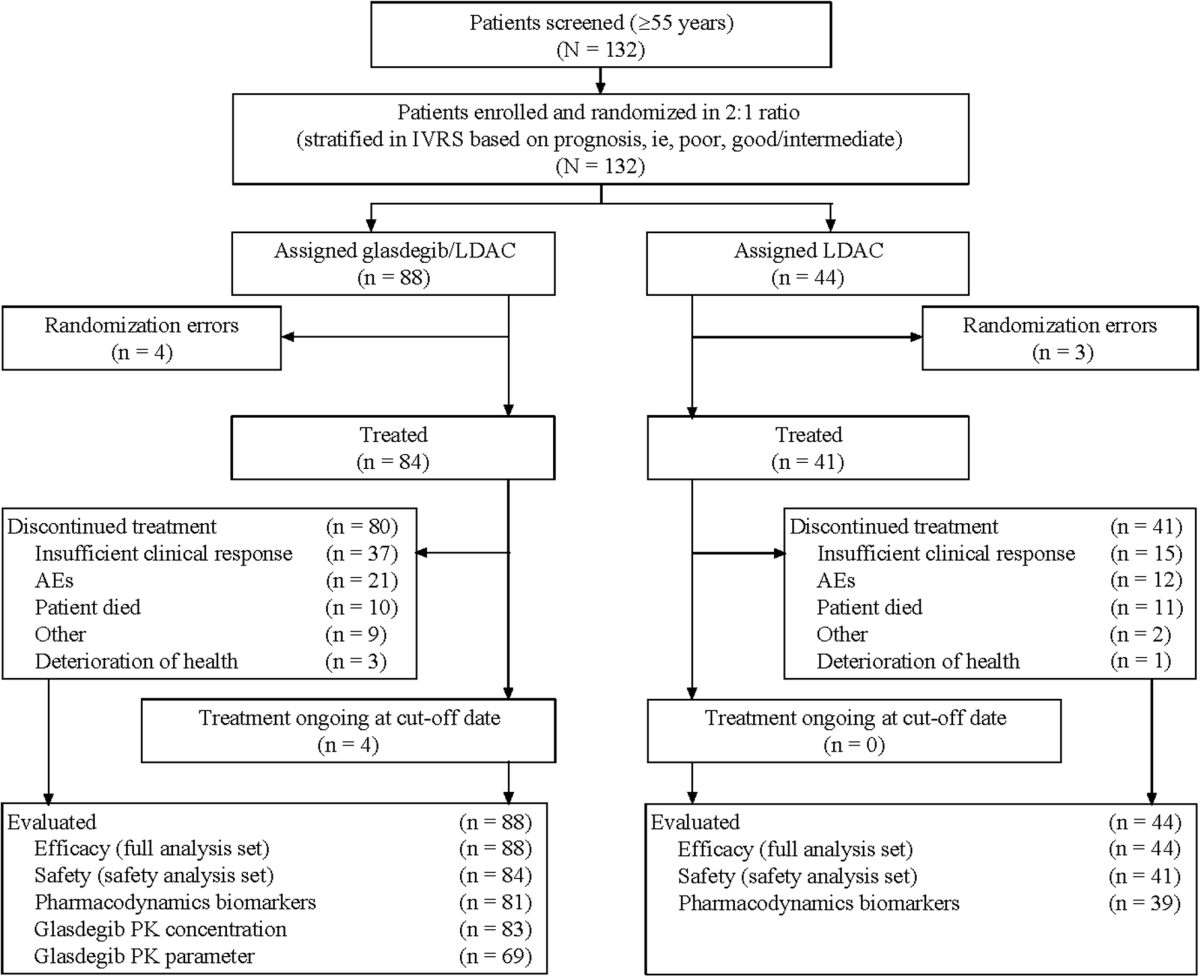
Patient disposition. This study is ongoing; the first patient randomization visit took place on 3 January 2014, and the primary analysis data cutoff was 3 January 2017. The randomization errors in 7/132 patients (5%) were due to patients withdrawing consent or failing to maintain eligibility requirements. Discontinuations were attributed to the last study treatment received. Treated was defined as patients who received at least one non-zero dose of glasdegib or LDAC. *AE* adverse event, *IVRS* interactive voice response system, *LDAC* low-dose cytarabine, *PK* pharmacokinetic(s)

Overall, 132 patients were randomized to receive glasdegib/LDAC (*n* = 88) and LDAC (*n* = 44); among them, 84 and 41 patients received study treatments, respectively (Fig. 1). Patient demographic and baseline characteristics are summarized in Table 1. More male patients were included (69 in the glasdegib/LDAC and 26 in the LDAC group) and over half of the patients in each group (53/88 [60.2%] in the glasdegib/LDAC and 24/44 [54.5%] in the LDAC group) were aged >75 years. The median (range) number of cycles administered was 3 (1–35) with glasdegib/LDAC and 2 (1–9) with LDAC. Among patients who received treatment, 37/84 (44%) patients in the glasdegib/LDAC group and 15/41 (36.6%) patients in the LDAC group received follow-up systemic therapies after discontinuation of the study treatment. The majority of patients (34/84 [40.5%] in the glasdegib/LDAC and 14/41 [34.1%] in the LDAC group) received chemotherapy, primarily hypomethylating agents or palliative chemotherapy (Table S1).

**Table 1:** Patient demographic and baseline characteristics

	Glasdegib 100 mg+LDAC, *N* = 88	LDAC, *N* = 44
Sex, *n* (%)		
Female	19 (21.6)	18 (40.9)
Male	69 (78.4)	26 (59.1)
Age (years), *n* (%)		
55–64	2 (2.3)	1 (2.3)
65–74	33 (37.5)	19 (43.2)
≥75	53 (60.2)	24 (54.5)
Mean (SD)	76.2 (6.2)	74.5 (4.9)
Median (range)	77 (63–92)	75 (58–83)
Race, *n* (%)		
White	85 (96.6)	44 (100.0)
Black	1 (1.1)	0
Asian	2 (2.3)	0
Body mass index (kg/m^2^)		
Mean (SD)	27.4 (4.2)	28.2 (5.5)
Range	17.5–41.9	20.0–48.2
Peripheral blood white cell count (10^3^/mm^3^)		
Median (range)	2.3 (0.6–64.0)	3.6 (1.1–45.2)
Diagnosis^a^, *n* (%)		
AML	78 (88.6)	38 (86.4)
MDS	10 (11.4)	6 (13.6)
Bone marrow blasts (%)		
With AML, median (range)	41.0 (16.0–100.0)	46.0 (13.0–95.0)
With MDS, median (range)	14.0 (7.5–18.0)	16.0 (10.5–19.0)
Duration since histopathological diagnosis (months)		
AML, median (range)	0.6 (0.03–3.52)	0.5 (0.07–3.84)
MDS, median (range)	1.0 (0.20–13.63)	2.2 (0.43–14.98)
ECOG performance status, *n* (%)		
0	11 (12.5)	3 (6.8)
1	29 (33.0)	18 (40.9)
2	47 (53.4)	23 (52.3)
Not reported	1 (1.1)	0
Cytogenetic risk^b^, *n* (%)		
Good/intermediate risk	52 (59.1)	25 (56.8)
Poor risk	36 (40.9)	19 (43.2)
ELN risk stratification for AML [21], *n* (%)	*N* = 78	*N* = 38
Favorable	5 (6.4)	3 (7.9)
Intermediate-I	27 (34.6)	11 (28.9)
Intermediate-II	21 (26.9)	8 (21.1)
Adverse	25 (32.1)	16 (42.1)
Prognostic factors for MDS^c^, *n* (%)	*N* = 10	*N* = 6
Good risk	3 (30.0)	2 (33.3)
Intermediate risk	1 (10.0)	3 (50.0)
Poor risk	6 (60.0)	1 (16.7)
MDS IPSS score [22], *n* (%)	*N* = 10	*N* = 6
0.5–1 (Intermediate-1)	0	2 (33.3)
1.5–2 (Intermediate-2)	4 (40.0)	4 (66.7)
≥2.5 (High)	6 (60.0)	0
Prior therapy with MDS drug^d^, *n* (%)	*N* = 88	*N* = 44
Azacitidine	13 (14.8)	8 (18.2)
Decitabine	2 (2.3)	1 (2.3)

*AHD* antecedent hematologic disease, *AML* acute myeloid leukemia, *CR* complete remission or complete response, *ECOG* Eastern Cooperative Oncology Group, *HMA* hypomethylating agents, *IPSS* International Prognostic Scoring System, *LDAC* low-dose cytarabine, *MDS* myelodysplastic syndrome, *SD* standard deviation

^a^Secondary AML included AML evolving from MDS or other AHD and AML after previous cytotoxic therapy or radiation. Secondary MDS included MDS from prior AHD

^b^For AML, good/intermediate cytogenetic risk = favorable, intermediate-I, and intermediate-II risk groups; poor cytogenetic risk = adverse risk group

^c^MDS risk was assessed by cytogenetic abnormalities that were known at the time the study was initiated; good/intermediate cytogenetic risk = good and intermediate risk groups; poor cytogenetic risk = poor risk group

^d^All patients who received prior HMA therapy were considered refractory

### Efficacy

**Fig. 2:**
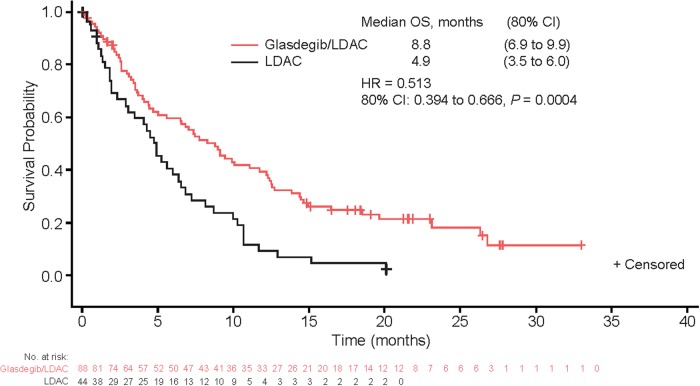
Kaplan–Meier estimate of overall survival, full analysis set. *CI* confidence interval, *HR* hazard ratio, *LDAC* low-dose cytarabine, *OS* overall survival

**Fig. 3:**
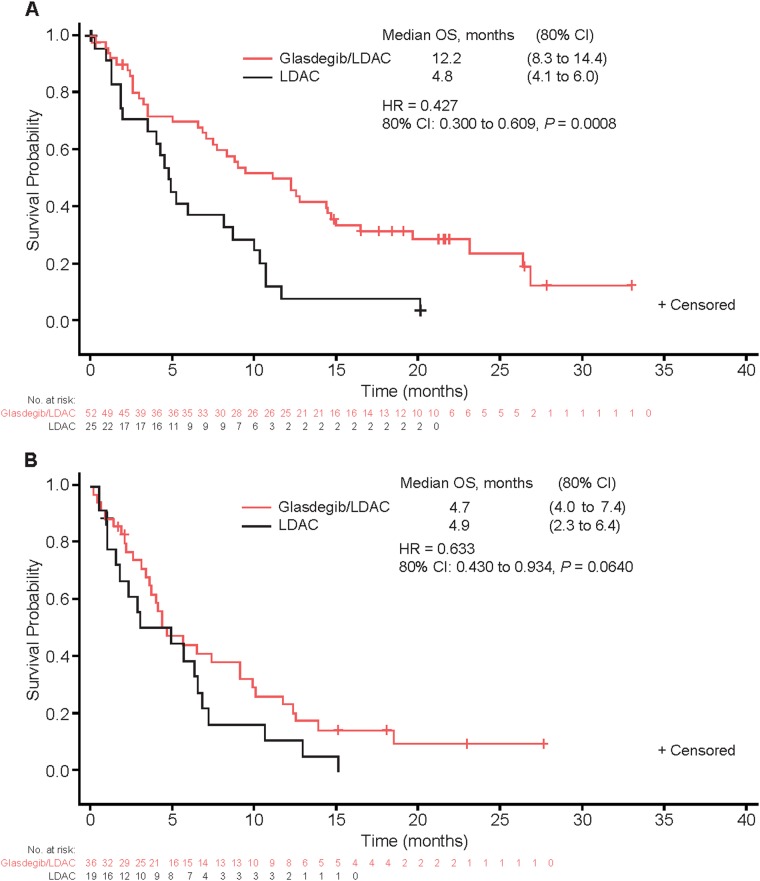
Kaplan-Meier estimate of overall survival, full analysis set, in patients at **A** good/intermediate cytogenetic risk and **B** poor cytogenetic risk. *CI* confidence interval, *HR* hazard ratio, *LDAC* low-dose cytarabine, *OS* overall survival

Median follow-up for OS was 21.7 months with glasdegib/LDAC and 20.1 months with LDAC. The corresponding number of deaths were 68/88 (77.3%) and 41/44 (93.2%) patients. The main cause of death in both arms was disease progression (Tables S2 and S3). This translated into a median (80% CI) OS of 8.8 (6.9–9.9) months with glasdegib/LDAC and 4.9 (3.5–6.0) months with LDAC (HR, 0.51 [80% CI, 0.39–0.67], *P* = 0.0004) (Fig. 2). The probability (80% CI) of being alive at 6 and 12 months, respectively, was 59.8% (52.6–66.3) and 39.5% (32.6–46.3) with glasdegib/LDAC versus 38.2% (28.6–47.7) and 9.5% (4.8–16.3) with LDAC. Results were similar when separate Cox proportional hazards model were estimated by cytogenetic risk (Fig. 3). In patients with AML (*n* = 116), median (80% CI) OS was 8.3 (6.6–9.5) months with glasdegib/LDAC and 4.3 (2.9–4.9) months with LDAC (HR, 0.46 [80% CI, 0.35–0.62], *P* = 0.0002). In patients with MDS (*n* = 16), median (80% CI) OS was 10.9 (1.6–12.5) months with glasdegib/LDAC and 10.3 (6.0–11.7) months with LDAC (HR, 0.77 [80% CI, 0.37–1.63], *P* = 0.3280).

Fifteen of 88 (17.0%) patients in the glasdegib/LDAC arm and 1/44 (2.3%) patient in the LDAC arm achieved CR (*P* < 0.05, Table 2). In the glasdegib/LDAC arm, median (range) duration of response was 9.9 (0.03–28.8) months for patients with CR and 6.5 (0.03–28.8) months for patients with either CR, CRi, or MLFS. In the AML population, overall response rate (ORR; defined as CR plus CRi plus MLFS) was 26.9% (21/78) with glasdegib/LDAC and 5.3% (2/38) with LDAC. In the MDS population, ORR (defined as CR plus mCR) was 20.0% (2/10) with glasdegib/LDAC and 0% (0/6) with LDAC. Best overall response with other responses of interest for patients with AML and MDS are summarized in Tables S4 to S6.

**Table 2:** Proportion of patients with investigator-reported CR, full analysis set

	Glasdegib 100 mg+LDAC, *N* = 88	LDAC, *N* = 44
Patients with CR, *n* (%)	15 (17.0)	1 (2.3)
80% CI^a^	11.9–22.2	0.0–5.2
Cytogenetic risk		
Good/intermediate	52	25
Patients with CR, *n* (%)	10 (19.2)	0 (0.0)
80% exact CI^b^	12.3–28.1	0.0–8.8
Poor cytogenetic risk	36	19
Patients with CR, *n* (%)	5 (13.9)	1 (5.3)
80% exact CI^b^	6.9–24.2	0.6–19.0
Combination versus LDAC		
Pearson Chi-square test for all enrolled patients (unstratified)		
* P* value		0.0142
CMH test for all enrolled patients stratified by cytogenetics^c^		
Odds ratio (80% CI)		5.03 (1.59–15.88)
* P* value		0.0152

*CI* confidence interval, *CMH* Cochran–Mantel–Haenszel, *CR* complete remission, *IVRS* interactive voice response system, *LDAC* low-dose cytarabine

^a^Using normal approximation

^b^Using exact method based on binomial distribution

^c^Good/intermediate and poor cytogenetic risk based on IVRS

### Pharmacokinetics

Eighty-three and 69 patients in the glasdegib/LDAC arm were analyzed for PK concentration and PK parameters, respectively. Sixty-one of 69 patients evaluable for PK parameters were analyzed on Cycle 1 Day 10; of these, 41 did not receive cytochrome P450 (CYP) 3A4 (CYP3A4) inhibitors concomitantly. Since CYP3A4 inhibitors have the potential to increase glasdegib plasma exposure, this group was considered to more accurately represent glasdegib plasma PK parameters for the 100-mg once-daily dose. These patients showed a somewhat lower exposure to glasdegib than those with exposure to CYP3A4 inhibitors. Summary of glasdegib PK parameters for glasdegib/LDAC arm on Cycle 1 Day 10 is presented in Table S7. Median glasdegib plasma concentration–time profile on Cycle 1 Day 10 is presented in Fig. S1.

### Safety

The median (range) treatment duration was 2.7 (0.1–31.9) months with glasdegib/LDAC and 1.5 (0.2–7.9) months with LDAC. The mean relative dose intensity (for calculations, see Supplementary materials) of glasdegib was 89.0% for the glasdegib/LDAC arm, and the mean relative LDAC dose intensity was 95.5% and 96.1% for the glasdegib/LDAC and LDAC arms, respectively.

The most frequently (>5% of patients) reported nonhematologic grade 3/4 all-causality AEs with glasdegib/LDAC were pneumonia (16.7% [14/84]), fatigue (14.3% [12/84]), dyspnea (7.1% [6/84]), hyponatremia, sepsis, and syncope (6.0% [5/84], each), and pneumonia (14.6% [6/41]) with LDAC (Table 3). The most frequently (>5% of patients) reported nonhematologic grade 3/4 treatment-related AE (i.e., related to either LDAC and/or glasdegib) was fatigue (10.7% [9/84]), which occurred in the glasdegib/LDAC arm (Table S8).

**Table 3:** Treatment-emergent all-causality adverse events occurring in ≥20% of patients in any treatment

MedDRA preferred term^a^, *n* (%)	Glasdegib 100 mg+LDAC, *N* = 84				LDAC, *N* = 41			
	Grade 1–2	Grade 3–4	Grade 5	Total	Grade 1–2	Grade 3–4	Grade 5	Total
Any AEs	6 (7.1)	54 (64.3)	24 (28.6)	84 (100.0)	1 (2.4)	23 (56.1)	17 (41.5)	41 (100.0)
Anemia	3 (3.6)	35 (41.7)	0	38 (45.2)	2 (4.9)	15 (36.6)	0	17 (41.5)
Febrile neutropenia	0	30 (35.7)	0	30 (35.7)	0	10 (24.4)	0	10 (24.4)
Nausea	28 (33.3)	2 (2.4)	0	30 (35.7)	4 (9.8)	1 (2.4)	0	5 (12.2)
Decreased appetite	25 (29.8)	3 (3.6)	0	28 (33.3)	3 (7.3)	2 (4.9)	0	5 (12.2)
Fatigue	14 (16.7)	12 (14.3)	0	26 (31.0)	6 (14.6)	2 (4.9)	0	8 (19.5)
Thrombocytopenia	0	26 (31.0)	0	26 (31.0)	1 (2.4)	10 (24.4)	0	11 (26.8)
Pneumonia	4 (4.8)	14 (16.7)	6 (7.1)	24 (28.6)	1 (2.4)	6 (14.6)	3 (7.3)	10 (24.4)
Diarrhea	19 (22.6)	4 (4.8)	0	23 (27.4)	8 (19.5)	1 (2.4)	0	9 (22.0)
Pyrexia	21 (25.0)	2 (2.4)	0	23 (27.4)	7 (17.1)	2 (4.9)	0	9 (22.0)
Edema peripheral	22 (26.2)	0	0	22 (26.2)	6 (14.6)	1 (2.4)	0	7 (17.1)
Constipation	20 (23.8)	1 (1.2)	0	21 (25.0)	6 (14.6)	0	0	6 (14.6)
Dysgeusia	21 (25.0)	0	0	21 (25.0)	1 (2.4)	0	0	1 (2.4)
Dyspnea	15 (17.9)	6 (7.1)	0	21 (25.0)	9 (22.0)	2 (4.9)	0	11 (26.8)
Muscle spasms	15 (17.9)	4 (4.8)	0	19 (22.6)	2 (4.9)	0	0	2 (4.9)
Cough	18 (21.4)	0	0	18 (21.4)	6 (14.6)	1 (2.4)	0	7 (17.1)
Dizziness	17 (20.2)	1 (1.2)	0	18 (21.4)	4 (9.8)	0	0	4 (9.8)
Vomiting	16 (19.0)	2 (2.4)	0	18 (21.4)	3 (7.3)	1 (2.4)	0	4 (9.8)

*AE* adverse event, *LDAC* low-dose cytarabine, *MedDRA* Medical Dictionary for Regulatory Activities

^a^MedDRA (version 19.1) coding dictionary applied

Thirty of 84 (35.7%) and 19/41 (46.3%) patients permanently discontinued study treatments due to AEs, with 9/84 (10.7%) and 3/41 (7.3%) patients discontinuing due to treatment-related (per investigator’s assessment) AEs in the glasdegib/LDAC and LDAC arms, respectively. In the glasdegib/LDAC arm, 47/84 (56.0%) patients temporarily discontinued glasdegib and/or LDAC and 22/84 (26.2%) patients had study treatment dose reduced owing to AEs. In the LDAC arm, 13/41 (31.7%) patients temporarily discontinued LDAC due to AEs. No dose reduction in LDAC due to AEs was reported.

Serious AEs were reported in 66/84 (78.6%) patients in the glasdegib/LDAC arm and 32/41 (78.0%) patients in the LDAC arm. The most frequently (≥15% of patients) reported serious AEs were febrile neutropenia (28.6% [24/84] with glasdegib/LDAC, 17.1% [7/41] with LDAC) and pneumonia (22.6% [19/84] and 17.1% [7/41], respectively). In the glasdegib/LDAC arm, 3/84 (3.6%) patients had serious acute kidney injury (1 considered related to glasdegib) and 1/84 (1.2%) patient had serious muscle spasms (considered related to glasdegib).

Nine and five patients in the glasdegib/LDAC and LDAC arms, respectively, had elevated liver function parameters (total bilirubin, aspartate aminotransferase, and/or alanine aminotransferase). Most were grade 1/2; 3 patients in the glasdegib/LDAC arm had grade 3 (1 related and 2 unrelated to treatment). No patient had concurrent elevations of all enzymes and none was confirmed as Hy’s law case [25]. No elevated liver enzymes led to permanent discontinuations of study treatments.

Abnormal Frederica’s QTc (QTcF) findings, either mean QTcF >480 ms and/or mean QTcF increase >60 ms from baseline, occurred in 9 patients treated with glasdegib/LDAC and 5 treated with LDAC. QTcF prolongation >500 ms was less frequent with glasdegib/LDAC versus LDAC (6.0% [5/83] versus 11.8% [2/17]). Two patients temporarily discontinued treatment due to glasdegib-related electrocardiogram QT prolongation. Two patients had permanent dose reduction due to treatment-related electrocardiogram QT prolongation, 1 of which was related to glasdegib. No patients had Torsades de Pointes.

### Biomarker analyses

Eighty-eight patients were included in baseline mutational analyses of bone marrow and/or peripheral blood, including 61 patients who received glasdegib/LDAC and 27 patients who received LDAC. No significant differences in mutational frequency between responding and non-responding patients were evident (Fisher’s exact test, *P* > 0.05 for each of the 12 genes analyzed). Responses were observed in patients bearing mutations in ≥1 of all 12 genes assessed except *KRAS*, but the small numbers preclude firm conclusions of associations of mutations in specific genes with response to therapy (Table S9). However, nonsignificant trends suggest that gene mutations associated with a favorable overall response to the combination treatment include *CEBPA*, *IDH1*, *NPM1*, *RUNX1*, and *TET2*, whereas gene mutations associated with an unfavorable overall response to the combination treatment include *DNMT3A*, *IDH2*, and *NRAS/KRAS*. Further, an ad hoc exploratory analysis demonstrated no significant relationship to response for *TP53* mutational status (data not shown). Findings of RNA biomarker analysis are described in Supplementary materials.

## Discussion

This randomized phase II trial in patients with AML or high-risk MDS met its primary endpoint, as the addition of glasdegib to LDAC demonstrated statistically significant and clinically meaningful OS improvement. The patients treated with glasdegib/LDAC achieved a 49% reduction in the risk of death relative to LDAC (median 8.8 versus 4.9 months; HR, 0.51 [80% CI, 0.39 to 0.67], *P* = 0.0004). In terms of the HR, improvement in OS was consistent across pre-specified subgroups by cytogenetic risk, particularly in patients with good/intermediate cytogenetic risk. Furthermore, ORR with glasdegib/LDAC (26.9%) was higher compared with LDAC (5.3%). These results, together with the manageable safety profile, make the combination of Hedgehog inhibition with LDAC a compelling therapeutic approach particularly for patients with AML ineligible for intensive chemotherapy.

The subset of MDS patients treated with glasdegib/LDAC achieved a 22.8% reduction in the risk of death relative to LDAC, though the 80% CI around the OS HR encompassed one and the sample size was small. Considering that the analysis of patients with MDS was limited by the small sample size, more patients with MDS are being assessed (ClinicalTrials.gov, NCT02367456) to better understand the impact of glasdegib in MDS.

A median of two cycles of LDAC was administered, which was a shorter treatment period than the four cycles delivered in a prior study [26]. The open-label design of the current study may have contributed to this short treatment period with LDAC; however, this median number of cycles of LDAC was consistent with a most recent report by Dennis et al. [27]. The CR rate in patients treated with glasdegib/LDAC (17.0%) was higher than in those treated with LDAC (2.3%). These results showed a lower CR rate with LDAC than previously published (7–22%), potentially because of the short treatment period (1.5 months) with LDAC in the current study [2, 5–7, 26, 28, 29]. The low CR rate in the LDAC arm in the current study may also be due in part to the high proportion of patients with secondary AML who are known to be resistant to chemotherapy [30]. However, median OS with LDAC was similar to that observed in previous studies, suggesting that the control arm is representative of clinical expectations with this regimen [6, 31, 32].

In the population treated with glasdegib/LDAC, glasdegib mean steady-state plasma PK parameters at 100 mg once daily were in agreement with the mean parameters observed in the phase Ib portion (Arm A) of the study [17]. The maximum plasma concentration (*C*_max_) of glasdegib at 100 mg is adequate to cover the half maximal inhibitory concentration values required for inhibition of the Hedgehog pathway in vitro [16]. The similar means of *C*_max_ and AUC_tau_ and the variability in these parameters (range, 44–61%) suggest that the intermittent use of moderate or strong CYP3A4 inhibitors is not associated with a large increase in glasdegib exposures on Cycle 1 Day 10. This indicates that CYP3A4 inhibitors may be used concomitantly as medically necessary.

Although comparison between trials should be considered with caution due to potential methodologic and other differences, median OS with glasdegib/LDAC compared favorably to previously reported outcomes with the combinations of LDAC/imatinib (4.6 months), LDAC/lintuzumab (4.7 months), or LDAC/volasertib (phase II 8.0 months, phase III 4.8 months) [6, 31–33]. Importantly, the addition of glasdegib to LDAC was generally well tolerated, with a manageable safety profile consistent with elderly patients receiving chemotherapy and toxicities reported for other marketed Smoothened inhibitors. The frequencies of alopecia, muscle spasms, and dysgeusia were numerically lower than what has been previously reported for Smoothened inhibitors [34–36]. The most common AEs occurring at higher rates in the glasdegib/LDAC versus LDAC arm were cytopenias and gastrointestinal events (mostly grade 1–2). Cytopenias were not accompanied by increases in sepsis or bleeding as compared with LDAC. Patients in the glasdegib/LDAC arm remained longer on treatment compared with the LDAC arm; therefore, it is possible the higher incidence of cytopenias in the glasdegib/LDAC arm was due to the longer duration of chemotherapy.

Preliminary signs of clinical efficacy were evident across patients with diverse mutational profiles, suggesting the potential for broad efficacy of glasdegib in combination with LDAC. However, no significant correlations were evident between mutational status of any of the individual 12 reported genes and clinical response. Nonsignificant trends suggesting association of gene mutations with response or lack of response were noted, but further research is required.

Reducing the incidence of disease progression to prolong survival remains the highest unmet medical need in the treatment of AML. Various agents targeting distinct pathways or markers are currently in development or have become available for clinical management of AML, such as azacitidine and venetoclax. Both drugs showed promising effects in treating AML as debulking agents [37, 38] via a different mechanism than that of the stem cell agent glasdegib. Preclinical data showed synergistic activity of Smoothened inhibitor (erismodegib) and azacitidine [39], and in a phase I trial glasdegib plus azacitidine showed evidence of clinical activity with no evidence of drug–drug interaction [40]. Aiming for an effective assessment of a novel therapy for patients with AML, a randomized, double-blind, multicenter, placebo controlled phase III trial (ClinicalTrials.gov, NCT03416179) of glasdegib in combination with intensive chemotherapy or azacitidine in patients with untreated AML is ongoing.

The addition of glasdegib to LDAC resulted in a favorable benefit-to-risk profile given the statistically significant and clinically meaningful improvement in OS compared with the standard therapy of LDAC and generally manageable toxicity. Therefore, the combination of glasdegib plus LDAC may represent a promising treatment strategy for patients with AML or high-risk MDS who are not suitable for intensive chemotherapy.

## Supplementary information

Supplemental material
