# Supplementary material for: Randomized comparison of low dose cytarabine with or without glasdegib in patients with newly diagnosed acute myeloid leukemia or high-risk myelodysplastic syndrome
Source: Leukemia. 2018 Dec 16;33(2):379–89. doi: 10.1038/s41375-018-0312-9 (PMC6365492; doi:10.1038/s41375-018-0312-9)
Supplement: Supplementary file 1 — Supplemental material [file 41375_2018_312_MOESM1_ESM.docx]

**Supplementary Materials**

**Randomized Comparison of Low Dose Cytarabine With or Without Glasdegib in Patients With Newly Diagnosed Acute Myeloid Leukemia or High-Risk Myelodysplastic Syndrome**

Jorge E. Cortes, Florian H. Heidel, Andrzej Hellmann, Walter Fiedler, B. Douglas Smith, Tadeusz Robak, Pau Montesinos, Daniel A. Pollyea, Pierre DesJardins, Oliver Ottmann, Weidong Wendy Ma, M. Naveed Shaik, A. Douglas Laird, Mirjana Zeremski, Ashleigh O’Connell, Geoffrey Chan, Michael Heuser

Table of Contents

[Methods 1](#_Toc528326660)

[Patients 1](#_Toc528326661)

[Inclusion Criteria 2](#_Toc528326662)

[Assessments 5](#_Toc528326663)

[Statistical Analyses 6](#_Toc528326664)

[Results 7](#_Toc528326665)

[Findings of RNA Biomarker Analysis 7](#_Toc528326666)

[Figure S1. Median glasdegib plasma concentration–time profile on Cycle 1 Day 10, semi-log 7](#_Toc528326667)

[Table S1. Follow-up systemic therapies in all patients who received glasdegib 100 mg plus LDAC and LDAC only 8](#_Toc528326668)

[Table S2. Deaths within 30 days and 60 days of treatment initiation 9](#_Toc528326669)

[Table S3. All-causality treatment-emergent SAEs by MedDRA preferred term in ≥2 patients 10](#_Toc528326670)

[Table S4: Investigator-reported best overall response for patients with AML 11](#_Toc528326671)

[Table S5. Investigator-reported best overall response for patients with MDS 12](#_Toc528326672)

[Table S6. Patients who were not evaluable for disease response 13](#_Toc528326673)

[Table S7. Glasdegib plasma pharmacokinetic parameters on Cycle 1 Day 10 14](#_Toc528326674)

[Table S8. Treatment-related all causality AEs occurring in ≥10% of patients in any treatment arm 15](#_Toc528326675)

[Table S9. Baseline gene mutation frequency in responding patients by treatment arm 16](#_Toc528326676)

# Methods

## Patients

### Inclusion Criteria

Patient eligibility should be reviewed and documented by an appropriately qualified member of the investigator’s study team before patients are included in the study.

Patients must meet all of the following inclusion criteria to be eligible for enrollment into the study:

1. Patients with acute myeloid leukemia (AML) or refractory anemia with excess blasts (RAEB)-2 high-risk myelodysplastic syndrome (MDS) who are newly diagnosed according to the World Health Organization (WHO) 2008 Classification^[[1]](#footnote-1)^ and previously untreated. Eligible patients with MDS, as well as eligible patients with AML arising from an antecedent hematologic disease (AHD) or MDS, may have had **one** prior regimen with commercially-available agent(s) (e.g., azacitidine or decitabine) for the treatment of their prior hematologic disease. The patients may not have had any prior therapy for their AML.
2. Patients must have a known cytogenetic profile at study entry.
3. AML patients include de-novo AML, AML evolving from MDS or other AHD and AML after previous cytotoxic therapy or radiation (secondary AML).
   - For a diagnosis of AML, a bone marrow blast count of 20% or more is required.
   - For AML defined by cytogenetic aberrations t(8;21), inv(16) or t(16;16) and some cases of erythroleukemia the proportion of bone marrow blasts may be <20%.
   - In AML French-American-British (FAB) M6a (erythroid leukemia) ≥20% of non-erythroid cells in the bone marrow must be leukemic blasts and ≥50% of the cells are erythroid precursors.
   - In AML with monocytic or myelomonocytic differentiation, monoblasts and promonocytes, but not abnormal monocytes, are counted as blast equivalents.
4. For a diagnosis of high-risk MDS RAEB-2 the patient must have 10-19% bone marrow blasts.
5. Age: must be ≥55 years old.
6. Eastern Cooperative Oncology Group (ECOG) Performance Status 0, 1, or 2.
7. Patients with AML or high-risk MDS who have **one or more** of the criteria below are considered unfit for intensive chemotherapy^[[2]](#footnote-2)^ and are eligible:
   - Age ≥75 years.
   - ECOG of 2.
   - Serum creatinine >1.3 mg/dL.
   - Severe cardiac disease (e.g., LVEF <45% by multi-gated acquisition [MUGA] or echocardiography [ECHO] at screening).
8. Adequate organ function as defined by the following:
   - Serum aspartate aminotransferase (AST) and serum alanine aminotransferase (ALT) ≤3 x upper limit of normal (ULN), or AST and ALT ≤5 x ULN if liver function abnormalities are due to underlying malignancy.
   - Total serum bilirubin ≤2 x ULN (except patients with documented Gilbert’s syndrome).
   - Serum creatinine ≤1.5 x ULN or estimated creatinine clearance ≥60 mL/min as calculated using the method standard for the institution.
9. All anti-cancer treatments (unless specified) should be discontinued ≥2 weeks from study entry, for example: targeted chemotherapy, radiotherapy, investigational agents, hormones, anagrelide or cytokines.
   - For control of rapidly progressing leukemia, hydroxyurea or leukopheresis may be used before and for up to 1 week after first dose of glasdegib.
   - Patients with controlled central nervous system (CNS) leukemia (documented by two consecutive assessments of zero blast count in cerebrospinal fluid), and who are still receiving intra-thecal (IT) therapy at study entry are considered eligible, and will continue to receive IT therapy.
10. Resolved acute effects of any prior therapy to baseline severity or Grade ≤1 Common Terminology Criteria for Adverse Events (CTCAE) except for AEs not constituting a safety risk by investigator judgement.
11. Serum/urine pregnancy test (for females of childbearing potential) that is negative at screening and immediately prior to initiation of treatment (first dose). Male and female patients of childbearing potential must agree to use a highly effective method of contraception throughout the study and for at least 180 days after the last dose of assigned treatment. A patient is of childbearing potential if, in the opinion of the investigator, he/she is biologically capable of having children and is sexually active.
12. Evidence of a personally signed and dated informed consent document indicating that the patient (or a legal representative) has been informed of all pertinent aspects of the study.
13. Willingness and ability to comply with the study scheduled visits, treatment plans, laboratory tests and other procedures.

Patients with leukocytes ≥30×10^9^/L at study entry were excluded; treatment with hydroxyurea or leukapheresis to reduce the leukocyte count below 30×10^9^/L prior to enrolment was permitted. Patients with active malignancy were excluded, with the exception of basal cell carcinoma, non-melanoma skin cancer, and cervical carcinoma in situ; other prior or concurrent malignancies were considered on a case-by-case basis. Other exclusion criteria included a recent myocardial infarction, congenital long QT syndrome, Torsades de Pointes, clinically significant ventricular arrhythmias within 6 months of study entry, or corrected QT (QTc) interval >470 ms using Fridericia’s formula (QTcF).

## Assessments

Samples for bone marrow evaluation were collected at screening, on Cycle 3 Day 1 and every third cycle, within 14 days of achieving initial hematologic recovery in the peripheral blood (defined as absolute neutrophil count >1000/µL and platelets ≥100,000/µL), end of treatment, and at the investigator discretion (±7 days of nominal time).

Blood samples for PK analysis of glasdegib were collected on Cycle 1 Day 1 at pre-dose and 1 and 4 hours post-dose; Cycle 1 Day 10 at pre-dose and 1, 2, 4, and 6 hours post-dose; and Cycles 2, 3, 4, and 5 on Day 1 at pre-dose, 1 and 4 hr post-dose.

Calibration standard responses were linear over the range of 0.2 ng/mL to 200 ng/mL for glasdegib, using a l/concentration^2^-weighted linear regression. The lower limit of quantification (LLOQ) for glasdegib was 0.2 ng/mL. Samples with plasma glasdegib concentrations below the LLOQ were reported as less than the LLOQ.

The inter-batch assay accuracy, expressed as percent relative error of the mean glasdegib quality control (QC) sample concentrations, ranged from –8.1% to 1.3%. Inter-batch assay precision, expressed as percent coefficient of variation (%CV) of the estimated glasdegib concentrations of QC samples, was ≤8.5%.

The pharmacokinetic (PK) parameters were calculated using noncompartmental analysis and included: maximum observed plasma concentration (C_max_), time to C_max_, area under the plasma concentration–time curve from time 0 to tau (tau *=* dosing interval of 24 hr), average plasma concentration at steady-state, and predose plasma concentration.

Treatment duration was calculated as the last dosing date of study drug minus Cycle 1 Day 1 plus 1 day, where last dosing date was the last non-zero dose date and it included missed doses on unknown dates. Time of treatment exposure of glasdegib was calculated as the last dosing date of study drug minus Cycle 1 Day 1 plus 1 day, where last dosing date was the last non-zero dose date and it excluded days with total dose administer of 0 mg.

Relative dose intensity was calculated as follows: relative dose intensity while on treatment *=* {actual total dose received/weeks from treatment start to end of treatment}/{planned intensity}, planned intensity *=* {initial planned dose/planned number of weeks in a cycle}, and actual cycle intensity *=* {actual received cycle dose/number of weeks in the cycle including delays}.

DNA samples extracted from peripheral blood or bone marrow were analyzed using next-generation DNA sequencing validated to Good Clinical Practice guidelines of a panel of 12 genes performed using the Illumina® MiSeq instrument (San Diego, CA, USA). In a secondary assay, an amplicon-based approach was used to further characterize the *FLT3* gene for the presence of internal tandem duplication (ITD) mutations. Whole blood samples from serial blood draws were analyzed for gene expression using TaqMan Low-Density Microarrays (TLDA). These TLDA cards included 21 target genes implicated in Smoothened pathway signaling and/or AML pathobiology, 2 endogenous control reference genes (*GUSB* and *TBP*), and 1 manufacturing control gene (*GAPDH*). The subset of time-points prioritized for gene expression and associated statistical analysis were short-term where blast counts were generally not substantially different compared with baseline, or were at end of treatment when blast counts had often rebounded.

## Statistical Analyses

A total of 92 overall survival (OS) events were needed to provide 80% power to detect a difference between the two arms. This was based on 2:1 randomization, a planned accrual period of approximately 13 months, a follow-up period of approximately 6 months, a one-sided log-rank test with alpha *=* 0.1 (type I error), and one futility analysis when 46 OS events were observed (50% information, rho[1] beta spending function).

# Results

## Findings of RNA Biomarker Analysis

RNA biomarkers were analyzed in 64 patients (47 and 17 patients in the glasdegib/LDAC and LDAC arms, respectively). Of the 21 mRNAs evaluated, several in the glasdegib/LDAC arm exhibited significant changes from baseline to end of treatment, including *CCND1* (median 60% lower than baseline, *P =* 0*.*0448), *CCND2* (median 30% lower than baseline, *P =* 0*.*0004), and *SMO* (median 60% lower than baseline, *P =* 0*.*0094). In the glasdegib/LDAC arm, response was associated with lower baseline *FOXM1* mRNA expression (median 50% of non-responders; *P =* 0*.*0258) and higher baseline *PTCH1* mRNA expression (median 2-fold higher than non-responders; *P =* 0*.*0002). Higher Cycle 1 Day 1 1h post-dose *MYCN* mRNA expression was associated with response (median ratio to baseline of 1.6 for responders relative to 0.5 for non-responders, *P =* 0*.*0312). Expression of mRNAs encoding the GLI1 and GLI2 transcription factors (Hedgehog pathway dependent transcripts) did not prove evaluable in almost all blood samples.

# Figure S1. Median glasdegib plasma concentration–time profile on Cycle 1 Day 10, semi-log

# Table S1. Follow-up systemic therapies in all patients who received glasdegib 100 mg plus LDAC and LDAC only

|  | **Glasdegib 100 mg + LDAC** | **LDAC** |
| --- | --- | --- |
| Total patients, n | 84 | 41 |
| With follow-up systemic therapies, n (%) | 37 (44.0) | 15 (36.6) |
| Transplant | 1 (1.2) | 0 |
| Chemotherapy^a^ | 34 (40.5) | 14 (34.1) |
| Biologic | 0 | 0 |
| Tyrosine kinase inhibitor | 0 | 0 |
| Investigational | 2 (2.4) | 0 |
| Other | 0 | 1 ( 2.4) |

^a^ Primarily hypomethylating agents or palliative chemotherapy (i.e., azacitidine, decitabine, hydroxyurea, and hydroxycarbamine).

Abbreviations: LDAC, low dose cytarabine.

# Table S2. Deaths within 30 days and 60 days of treatment initiation

|  | **Total** | | **AML** | | **MDS** | |
| --- | --- | --- | --- | --- | --- | --- |
|  | **Glasdegib 100 mg + LDAC**  **N *=* 84** | **LDAC**  **N *=* 41** | **Glasdegib 100 mg + LDAC**  **N *=* 75** | **LDAC**  **N *=* 36** | **Glasdegib 100 mg + LDAC**  **N *=* 9** | **LDAC**  **N *=* 5** |
| Deaths within 30 days | | | | | | |
| n (%) | 5 (6.0) | 5 (12.2) | 5 (6.7) | 5 (13.9) | 0 | 0 |
| 80% CI | 2.9–10.8 | 6.1–21.5 | 3.3–12.0 | 6.9–24.2 | 0 | 0 |
| Cause of death^a^ |  |  |  |  | 0 | 0 |
| Disease under study | 4 (4.8) | 4 (9.8) | 4 (5.3) | 4 (11.1) | 0 | 0 |
| Other | 1 (1.2) | 4 (9.8) | 1 (1.3) | 4 (11.1) | 0 | 0 |
| Deaths within 60 days | | | | | | |
| n (%) | 10 (11.9) | 13 (31.7) | 8 (10.7) | 13 (36.1) | 2 (22.2) | 0 |
| 80% CI | 7.5–17.7 | 22.1–42.8 | 6.3–16.7 | 25.3–48.1 | 6.1–49.0 | 0 |
| Cause of death^a^ |  |  |  |  |  |  |
| Disease under study | 9 (10.7) | 12 (29.3) | 7 (9.3) | 12 (33.3) | 2 (22.2) | 0 |
| Other | 3 (3.6) | 5 (12.2) | 2 (2.7) | 5 (13.9) | 1 (11.1) | 0 |

Patients could have multiple reasons for cause of death.

Abbreviations: AML, acute myeloid leukemia; CI, confidence interval; LDAC, low dose cytarabine; MDS, myelodysplastic syndrome.

# **Table S3.** All-causality treatment-emergent SAEs by MedDRA preferred term in ≥2 patients

|  | **Glasdegib 100 mg + LDAC, N *=* 84** | | | | | **LDAC Alone, N *=* 41** | | | |
| --- | --- | --- | --- | --- | --- | --- | --- | --- | --- |
| **MedDRA preferred term** | **Grade 2** | **Grade 3** | **Grade 4** | **Grade 5** | **Total** | **Grade 3** | **Grade 4** | **Grade 5** | **Total** |
| Any SAEs | 2 (2.4) | 28 (33.3) | 12 (14.3) | 24 (28.6) | 66 (78.6) | 9 (22.0) | 6 (14.6) | 17 (41.5) | 32 (78.0) |
| Febrile neutropenia | 0 | 20 (23.8) | 4 (4.8) | 0 | 24 (28.6) | 5 (12.2) | 2 (4.9) | 0 | 7 (17.1) |
| Pneumonia | 1 (1.2) | 10 (11.9) | 2 (2.4) | 6 (7.1)^a^ | 19 (22.6) | 2 (4.9) | 2 (4.9) | 3 (7.3) | 7 (17.1) |
| Disease progression | 0 | 0 | 0 | 8 (9.5) | 8 (9.5) | 0 | 0 | 5 (12.2) | 5 (12.2) |
| Anemia | 0 | 4 (4.8) | 2 (2.4) | 0 | 6 (7.1) | 0 | 0 | 0 | 0 |
| Syncope | 0 | 4 (4.8) | 0 | 0 | 4 (4.8) | 0 | 0 | 0 | 0 |
| Acute kidney injury | 1 (1.2) | 2 (2.4) | 0 | 0 | 3 (3.6) | 0 | 0 | 0 | 0 |
| Fatigue | 2 (2.4) | 1 (1.2) | 0 | 0 | 3 (3.6) | 0 | 0 | 0 | 0 |
| Hemorrhage intracranial | 0 | 0 | 2 (2.4) | 1 (1.2) | 3 (3.6) | 0 | 0 | 0 | 0 |
| Pyrexia | 2 (2.4) | 1 (1.2) | 0 | 0 | 3 (3.6) | 0 | 0 | 0 | 0 |
| Sepsis | 0 | 0 | 3 (3.6) | 0 | 3 (3.6) | 0 | 1 (2.4) | 4 (9.8)^b^ | 5 (12.2) |
| Cardiac arrest | 0 | 0 | 1 (1.2) | 1 (1.2) | 2 (2.4) | 0 | 0 | 0 | 0 |
| Cardiac failure | 0 | 0 | 2 (2.4) | 0 | 2 (2.4) | 0 | 0 | 0 | 0 |
| Fall | 1 (1.2) | 1 (1.2) | 0 | 0 | 2 (2.4) | 0 | 0 | 0 | 0 |
| Gastrointestinal hemorrhage | 0 | 1 (1.2) | 1 (1.2) | 0 | 2 (2.4) | 0 | 0 | 0 | 0 |
| Hyponatremia | 0 | 1 (1.2) | 1 (1.2) | 0 | 2 (2.4) | 0 | 0 | 0 | 0 |
| Muscular weakness | 0 | 2 (2.4) | 0 | 0 | 2 (2.4) | 0 | 0 | 0 | 0 |
| Myocardial infarction | 0 | 0 | 1 (1.2) | 1 (1.2) | 2 (2.4) | 0 | 0 | 0 | 0 |
| Septic shock | 0 | 0 | 1 (1.2) | 1 (1.2) | 2 (2.4) | 0 | 0 | 0 | 0 |
| Sudden death | 0 | 0 | 0 | 2 (2.4) | 2 (2.4) | 0 | 0 | 0 | 0 |
| Pancytopenia | 0 | 0 | 0 | 0 | 0 | 2 (4.9) | 0 | 0 | 2 (4.9) |

Values are n (%), from all cycles, safety analysis set.

Treatment-emergent AEs were defined as within 28 days of last dose of study treatment and graded in accordance with National Cancer Institute CTCAE version 4.03. Grade 5 is death related to AE. The type of Grade 5 events were characteristic of patients with acute myeloid malignancies, elderly patients, and chemotherapy treatment. No Grade 1 AEs were reported in ≥2 patients in either treatment arm. No Grade 2 AEs were reported in ≥2 patients in the LDAC arm.

^a^ One (1.2%) was considered as treatment-related Grade 5 AE.

^b^ One (2.4%) was treatment-related Grade 5 AE.

Abbreviations: AE, adverse event; CTCAE, Common Terminology Criteria for Adverse Events; LDAC, low-dose cytarabine; MedDRA, Medical Dictionary for Regulatory Activities; SAE, serious adverse event.

# Table S4: Investigator-reported best overall response for patients with AML

|  | **Glasdegib 100 mg + LDAC**  **N *=* 78** | | **LDAC**  **N *=* 38** | |
| --- | --- | --- | --- | --- |
|  | **n (%)** | **80% CI** | **n (%)** | **80% CI** |
| Objective response^a^ |  |  |  |  |
| Disease status |  |  |  |  |
| CR | 14 (17.9) | 12.4–24.8 | 1 (2.6) | 0.3–9.9 |
| CRi | 5 (6.4) | 3.2–11.6 | 1 (2.6) | 0.3–9.9 |
| MLFS | 2 (2.6) | 0.7–6.7 | 0 (0.0) | 0.0–5.9 |
| Not evaluable^b^ | 24 (30.8) | 23.9–38.4 | 16 (42.1) | 31.1–53.8 |
| ORR (CR+CRi+MLFS)^c^ | 21 (26.9) | 20.5–33.4 | 2 (5.3) | 0.6–9.9 |

From the full analysis set.

^a^ Using exact method based on binomial distribution and CIs are expressed in percentages.

^b^ In addition to the seven patients who were randomized but not treated, the majority of patients not evaluable for disease response in both arms were due to AE or patient died prior to on-study bone marrow evaluation.

^c^ Using normal approximation for further endpoints of interest and CIs are expressed in percentages.

Abbreviations: AML, acute myeloid leukemia; CI, confidence interval; CR, complete remission; CRi, complete remission with incomplete blood count recovery; LDAC, low dose cytarabine; MLFS, morphologic leukemia-free state; N, all treated patients; ORR, overall response rate.

# Table S5. Investigator-reported best overall response for patients with MDS^[[3]](#footnote-3)^

|  | **Glasdegib 100 mg + LDAC**  **N *=* 10** | | **LDAC**  **N *=* 6** | |
| --- | --- | --- | --- | --- |
|  | **n (%)** | **80% CI** | **n (%)** | **80% CI** |
| Objective response^a^ |  |  |  |  |
| Disease status |  |  |  |  |
| CR/Unconfirmed CR | 1 (10.0) | 1.0–33.7 | 0 (0.0) | 0.0–31.9 |
| PR/Unconfirmed PR | 0 (0.0) | 0.0–20.6 | 0 (0.0) | 0.0–31.9 |
| mCR/Unconfirmed mCR | 1 (10.0) | 1.0–33.7 | 0 (0.0) | 0.0–31.9 |
| Not evaluable^b^ | 2 (20.0) | 5.5–45.0 | 1 (16.7) | 1.7–51.0 |
| ORR (CR+mCR)^c^ | 2 (20.0) | 5.5–45.0 | 0 (0.0) | 0.0–31.9 |

From the full analysis set.

^a^ Using exact method based on binomial distribution and CIs are expressed in percentages.

^b^ On the glasdegib + LDAC arm, 1 patient did not receive study treatments and the other patient had adverse event prior to bone marrow evaluation; on the LDAC arm, the patient did not receive study treatment.

^c^ Using normal approximation for further endpoints of interest and CIs are expressed in percentages.

Abbreviations: CI, confidence interval; CR, complete remission; LDAC, low dose cytarabine; mCR, marrow complete remission; MDS, myelodysplastic syndrome; N, all treated patients; ORR, overall response rate; PR, partial remission.

# **Table S6.** Patients who were not evaluable for disease response

|  | **Glasdegib 100 mg + LDAC**  **N = 26** | **LDAC**  **N = 17** | **Total**  **N = 43** |
| --- | --- | --- | --- |
| Reasons for not being evaluable for bone marrow disease response, n (%) |  |  |  |
| AE leading to study termination prior to disease assessment | 9 (34.6) | 5 (29.4) | 14 (32.5) |
| Patient died prior to disease assessment | 3 (11.5) | 6 (35.3) | 9 (20.9) |
| Never started treatment | 4 (15.4) | 3 (17.6) | 7 (16.3) |
| Insufficient clinical response (based on peripheral blood only, no bone marrow performed) | 4 (15.4) | 2 (11.8) | 6 (14) |
| Patient refused prior to disease assessment (withdrew consent, local treatment, no further treatment, unwilling to comply) | 5 (19.2) | 1 (5.9) | 6 (14) |
| Global deterioration of health | 1 (3.9) | 0 | 1 (2.3) |

Abbreviations: AE, adverse event; LDAC, low dose cytarabine.

# **Table S7.** Glasdegib plasma pharmacokinetic parameters on Cycle 1 Day 10

| Parameter | Dose compliant, non-CYP3A4 N *=* 41^a^ | Dose compliant N *=* 61^b^ |
| --- | --- | --- |
| C_max_, ng/mL | 1252 (44) | 1343 (47) |
| T_max_, hr | 1.7 (0.67–5.8) | 2.0 (0.67–6.3) |
| AUC_tau_, ng·hr/mL^c^ | 17210 (54) | 19170 (61) |
| C_avg_, ng/mL | 718 (54) | 799 (61) |
| C_trough_, ng/mL | 427 (80) | 483 (88) |

For glasdegib 100 mg + LDAC arm.

Values are geometric mean (geometric % coefficient of variation) for all, except median (range) for T_max_.

^a^ n *=* 37 for AUC_tau_ and C_avg_; n *=* 36 for C_trough_.

^b^ n *=* 56 for AUC_tau_ and C_avg_; n *=* 55 for C_trough_.

^c^ For AUC_tau_, tau *=* 24 hr. For AUC_tau_, the pre-dose concentration was also designated as the 24-hr post-dose sample to estimate AUC_tau_, using assumption of steady state.

Definitions: C_max_, maximum observed plasma concentration; T_max_, time to first occurrence of C_max_; AUC_tau_, area under the plasma concentration-time profile from time 0 to tau (dosing interval, which was 24 hr); C_avg_, average plasma concentration at steady state; C_trough_, pre-dose plasma concentration.

Abbreviations: CYP, cytochrome P450; LDAC, low-dose cytarabine.

# Table S8. Treatment-related all causality AEs occurring in ≥10% of patients in any treatment arm

|  | **Glasdegib 100 mg + LDAC, N *=* 84** | | | **LDAC, N *=* 41** | | |
| --- | --- | --- | --- | --- | --- | --- |
| **MedDRA preferred term,* n (%)** | **Grade 1-2** | **Grade 3-5** | **Total** | **Grade 1-2** | **Grade 3-5** | **Total** |
| Any adverse event | 13 (15.5) | 55 (65.5) | 68 (81.0) | 10 (24.4) | 14 (34.1) | 24 (58.5) |
| Anemia | 4 (4.8) | 22 (26.2) | 26 (31.0) | 1 (2.4) | 5 (12.2) | 6 (14.6) |
| Nausea | 23 (27.4) | 1 (1.2) | 24 (28.6) | 1 (2.4) | 0 | 1 (2.4) |
| Decreased appetite | 19 (22.6) | 2 (2.4) | 21 (25.0) | 1 (2.4) | 1 (2.4) | 2 (4.9) |
| Thrombocytopenia | 0 | 20 (23.8) | 20 (23.8) | 0 | 5 (12.2) | 5 (12.2) |
| Dysgeusia | 19 (22.6) | 0 | 19 (22.6) | 0 | 0 | 0 |
| Fatigue | 10 (11.9) | 9 (10.7) | 19 (22.6) | 3 (7.3) | 1 (2.4) | 4 (9.8) |
| Muscle spasms | 13 (15.5) | 4 (4.8) | 17 (20.2) | 0 | 0 | 0 |
| Diarrhea | 11 (13.1) | 3 (3.6) | 14 (16.7) | 1 (2.4) | 0 | 1 (2.4) |
| Vomiting | 12 (14.3) | 2 (2.4) | 14 (16.7) | 3 (7.3) | 0 | 3 (7.3) |
| Platelet count decreased | 1 (1.2) | 12 (14.3) | 13 (15.5) | 0 | 1 (2.4) | 1 (2.4) |
| Febrile neutropenia | 0 | 12 (14.3) | 12 (14.3) | 0 | 3 (7.3) | 3 (7.3) |
| Weight decreased | 12 (14.3) | 0 | 12 (14.3) | 0 | 0 | 0 |
| White blood cell count decreased | 1 (1.2) | 10 (11.9) | 11 (13.1) | 1 (2.4) | 0 | 1 (2.4) |
| Constipation | 10 (11.9) | 0 | 10 (11.9) | 3 (7.3) | 0 | 3 (7.3) |
| Dyspnoea | 8 (9.5) | 2 (2.4) | 10 (11.9) | 1 (2.4) | 0 | 1 (2.4) |
| Neutrophil count decreased | 1 (1.2) | 9 (10.7) | 10 (11.9) | 0 | 1 (2.4) | 1 (2.4) |
| Alopecia | 9 (10.7) | 0 | 9 (10.7) | 0 | 0 | 0 |
| Neutropenia | 3 (3.6) | 6 (7.1) | 9 (10.7) | 0 | 4 (9.8) | 4 (9.8) |

Adverse events as related to either LDAC and/or glasdegib.

Abbreviations: AE, adverse event; LDAC, low-dose cytarabine; MedDRA, Medical Dictionary for Regulatory Activities.

# Table S9. Baseline gene mutation frequency in responding patients by treatment arm

| **Glasdegib 100 mg + LDAC**  **N *=* 61** | |  | **LDAC**  **N *=* 27** | |
| --- | --- | --- | --- | --- |
| Mutation | ORR, n (%) |  | Mutation | ORR, n (%) |
| *CEBPA*, n *=* 8 | 3 (38) |  | *CEBPA*, n *=* 3 | 0 |
| *DNMT3A*, n *=* 15 | 2 (13) |  | *DNMT3A*, n *=* 6 | 0 |
| *FLT3*, n *=* 5 | 1 (20) |  | *FLT3*, n *=* 0 | 0 |
| *FLT3-ITD*, n *=* 3 | 1 (33) |  | *FLT3-ITD*, n *=* 2 | 0 |
| *IDH1*, n *=* 10 | 5 (50) |  | *IDH1*, n *=* 2 | 0 |
| *IDH2*, n *=* 12 | 2 (17) |  | *IDH2*, n *=* 5 | 0 |
| *KIT*, n *=* 3 | 1 (33) |  | *KIT*, n *=* 1 | 0 |
| *KRAS*, n *=* 2 | 0 |  | *KRAS*, n *=* 2 | 0 |
| *NPM1*, n *=* 5 | 2 (40) |  | *NPM1*, n *=* 1 | 0 |
| *NRAS*, n *=* 5 | 1 (20) |  | *NRAS*, n *=* 3 | 0 |
| *RUNX1*, n *=* 28 | 10 (36) |  | *RUNX1*, n *=* 7 | 0 |
| *TET2*, n *=* 15 | 7 (47) |  | *TET2*, n *=* 9 | 1 (11) |
| *WT1*, n *=* 3 | 1 (33) |  | *WT1*, n *=* 1 | 0 |

The analysis population included patients with available sequencing results who were evaluable for response. Baseline mutational status determined from the combined results from evaluable bone marrow and/or whole blood samples. Mutational status assessed using next-generation sequencing (augmented by an amplicon-based assay in the case of *FLT3-ITD* mutations).

Statistical significance in comparison of responders with non-responders was determined using Fisher’s exact test. *P* > 0.30 for all evaluable comparisons.

For AML, investigator-reported ORR *=* CR+CRi+MLFS; for MDS, investigator-reported ORR *=* CR+mCR.

Abbreviations: AML, acute myeloid leukemia; CR, complete remission; CRi, complete remission with incomplete blood count recovery; LDAC, low-dose cytarabine; mCR, marrow complete remission; MDS, myelodysplastic syndrome; MLFS, morphologic leukemia-free state; ORR, overall response rate.

1. Vardiman JW, Thiele J, Arber DA *et al.* The 2008 revision of the World Health Organization (WHO) classification of myeloid neoplasms and acute leukemia: rationale and important changes. *Blood* 2009;114:937–51. [↑](#footnote-ref-1)
2. Kantarjian H, O'Brien S, Cortes J *et al.* Results of intensive chemotherapy in 998 patients age 65 years or older with acute myeloid leukemia or high-risk myelodysplastic syndrome: predictive prognostic models for outcome. *Cancer* 2006;106:1090–8. [↑](#footnote-ref-2)
3. Cheson BD, Greenberg PL, Bennett JM, et al. Clinical application and proposal for modification of the International Working Group (IWG) response criteria in myelodysplasia. *Blood* 2006;108:419–25. [↑](#footnote-ref-3)
